# Do Redox Balance and Inflammatory Events Take Place in Mild Bronchiectasis? A Hint to Clinical Implications

**DOI:** 10.3390/jcm10194534

**Published:** 2021-09-30

**Authors:** Liyun Qin, Maria Guitart, Mireia Admetlló, Sandra Esteban-Cucó, José María Maiques, Yingchen Xia, Jianhua Zha, Santiago Carbullanca, Xavier Duran, Xuejie Wang, Esther Barreiro

**Affiliations:** 1 Pulmonology Department-Muscle Wasting and Cachexia in Chronic Respiratory Diseases and Lung Cancer Research Group, IMIM-Hospital del Mar, Parc de Salut Mar, Health and Experimental Sciences Department (CEXS), Universitat Pompeu Fabra (UPF), Department of Medicine, Universitat Autònoma de Barcelona, Parc de Recerca Biomèdica de Barcelona (PRBB), 08003 Barcelona, Spain; liyun.qin@e-campus.uab.cat (L.Q.); mguitart@imim.es (M.G.); madmetllo@parcdesalutmar.cat (M.A.); 361439919013@email.ncu.edu.cn (Y.X.); 361439918044@email.ncu.edu.cn (J.Z.); Xuejie.Wang@e-campus.uab.cat (X.W.); 2 Centro de Investigación en Red de Enfermedades Respiratorias (CIBERES), Instituto de Salud Carlos III (ISCIII), 08003 Barcelona, Spain; 3 Laboratori de Referència de Catalunya, Clinical Microbiology and Parasitology Department, 08820 Barcelona, Spain; sestebanc@lrc.cat; 4 Radiology Department, Imatge Mèdica Intercentres-Parc de Salut Mar, Hospital del Mar, 08003 Barcelona, Spain; 40007@parcdesalutmar.cat (J.M.M.); 40163@parcdesalutmar.cat (S.C.); 5 Department of Thoracic Surgery, The First Affiliated Hospital of Nanchang University, Nanchang 330000, China; 6 Scientific and Technical Department, Hospital del Mar-IMIM, 08003 Barcelona, Spain; xduran@imim.es

**Keywords:** non-CF bronchiectasis patients, prooxidants, antioxidants, acute phase reactants, myeloperoxidase, reduced glutathione, bronchiectasis severity scores

## Abstract

We hypothesized that in mild bronchiectasis patients, increased systemic inflammation and redox imbalance may take place and correlate with clinical parameters. In plasma samples from patients with very mild bronchiectasis, inflammatory cells and molecules and redox balance parameters were analyzed. In the patients, lung function and exercise capacity, nutritional status, bacterial colonization, and radiological extension were assessed. Correlations between biological and clinical variables were determined. Compared to healthy controls, levels of acute phase reactants, neutrophils, IgG, IgA, myeloperoxidase, protein oxidation, and GSH increased and lung function and exercise capacity were mildly reduced. GSH levels were even greater in ex-smoker and *Pseudomona*-colonized patients. Furthermore, radiological extension inversely correlated with airway obstruction and, disease severity, and positively correlated with neutrophil numbers in mild bronchiectasis patients with no nutritional abnormalities. In stable patients with mild bronchiectasis, several important inflammatory and oxidative stress events take place in plasma. These findings suggest that the extension of bronchiectasis probably plays a role in the development of redox imbalance and systemic inflammation in patients with mild bronchiectasis. These results have therapeutic implications in the management of bronchiectasis patients.

## 1. Introduction

Non-cystic fibrosis (CF) bronchiectasis is a prevalent disease with a burden that is steadily increasing [1]. Bronchiectasis is a chronic respiratory condition characterized by the distortion of the airways, which favors favor the accumulation of lung secretions in patients. In the airways and lungs, bacterial colonization, chronic inflammation, and remodeling take place [2,3]. Acute exacerbations are also common in patients with bronchiectasis, thus, having a negative impact on their quality of life and disease prognosis. 

Inflammation and oxidative stress are mechanisms frequently observed in the airways and lungs of patients with bronchiectasis [4,5]. These are relevant mechanisms that underlie the pathophysiology of mucus hypersecretion and accumulation in the lungs and airways [6,7], which lead to further structural alterations in patients [6]. Oxidative stress modifies key cellular structures as a result of the action of oxidants on proteins, DNA, and lipids [8]. Inflammatory molecules through the action of cytokines may amplify the response to several insults in the lungs and airways of patients [9,10]. Most studies have focused on the examination of either antioxidant or prooxidant markers [10]. Increased oxidative stress levels are important players in the pathophysiology of skeletal muscle dysfunction and mass loss and nutritional abnormalities in patients with chronic obstructive pulmonary disease (COPD), especially those with more severe disease [11,12,13,14]. Whether oxidative stress may also play a substantial role in the development of systemic manifestations in other chronic respiratory diseases, even at early stages, remains to be answered. In this regard, in patients with mild bronchiectasis, the potential implications of an imbalance between oxidants and antioxidants still needs to be thoroughly analyzed. 

Systemic inflammation and oxidative stress have also been reported in patients with chronic respiratory diseases. COPD represents a paramount example of the presence of systemic inflammation and oxidative stress, particularly in patients with severe airflow limitation and emphysema and systemic effects of their respiratory condition (e.g., muscle dysfunction, sarcopenia, and poor exercise tolerance) [8,11,12,13,15,16]. In patients with mild-to-moderate COPD, the effects of systemic inflammation and/or redox imbalance are barely seen [13,16,17]. Likewise, in patients with bronchiectasis, greater systemic levels of inflammation are directly related to impaired lung function, quality of life, exercise tolerance, and higher scores of disease severity [10,18,19,20,21]. Whether patients with very mild bronchiectasis, localized extension, and preserved lung function as measured by several disease scores may also exhibit high levels of systemic inflammation and redox imbalance remains to be elucidated.

On this basis, we hypothesized that the systemic inflammatory profile and redox imbalance between prooxidants and antioxidants would differ in patients with very mild bronchiectasis from a group of healthy subjects. Hence, the study objectives were to thoroughly examine the following features: (1) systemic (blood) inflammatory cells and molecules, (2) systemic levels of prooxidants and antioxidants, (3) lung function and exercise capacity, (4) nutritional status, (5) bacterial colonization, and (6) correlations between clinical and biological variables in a prospective cohort of stable patients with mild bronchiectasis. A group of healthy subjects was also recruited for the purpose of comparisons. 

## 2. Methods

Full details on the different methodologies employed in the investigation are provided in the online Supplementary Materials. 

### 2.1. Study Subjects

This was a prospective, controlled, cross-sectional study, in which 30 patients (7 males) were recruited consecutively from the Multidisciplinary Bronchiectasis Unit of the Respiratory Department at Hospital del Mar (Barcelona, Spain) over the years 2019–2020. Additionally, 26 age- and sex-matched control subjects (9 males) were recruited from the general population (patients’ relatives or friends) at Hospital del Mar. In the patients, inclusion criteria were as follows: adults (18 years and over), diagnosis of non-CF bronchiectasis by high-resolution computerized tomography (HRCT) [22,23], no previous exacerbations of the disease at least 4 weeks prior to study entry (range from 2 months to four years). Exclusion criteria for all the patients and control subjects included other chronic cardiovascular or respiratory disorders, chronic metabolic diseases, signs of severe bronchial inflammation and/or infection, current or recent invasive mechanical ventilation, long-term oxygen therapy, and poor collaboration. Most of the patients recruited for the purpose of the investigation had a mild-to-moderate disease severity on the basis of lung function impairment, disease scoring using several indices, and radiologic extension [22,24,25,26,27]. All the patients were stable at the time of study entry. Approval was obtained from the institutional Ethics Committee on Human Investigation (Hospital del Mar-IMIM, Barcelona, Spain, protocol # 2019/8482/1, 14 March 2019) following the World Medical Association guidelines (Declaration of Helsinki, Fortaleza, Brazil, October 2013) for research on human beings. Informed written consent was obtained from all participants. 

### 2.2. Clinical Assessment

Nutritional evaluation included the assessment of body mass index (BMI) and determination of fat-free mass index (FFMI) using bioelectrical impedance [13,17,28,29,30]. Blood analytical parameters (systemic inflammatory cells and markers) were also obtained in all participants. Lung function parameters were determined in all study subjects following standard procedures [13,17,28,29,30]. Exercise capacity was assessed through the 6-min walking distance following previous methodologies [31]. Etiology of the non-CF bronchiectasis of all the study patients was also assessed.

#### 2.2.1. Bronchiectasis Severity Scores

The FACED (FEV_1_, AGE, Chronic colonization, Extension Dyspnea), EFACED (Exacerbation FACED), and BSI (Bronchiectasis Severity Index) scores were calculated in order to assess the clinical status and disease severity of the bronchiectasis patients according to previous reports [24,25,26]. 

#### 2.2.2. Radiological Extension

HRCT-scans were used to evaluate the radiological extension of bronchiectasis in all the study patients. Scores for each patient were calculated by two independent observers following previously established criteria [32,33]. 

#### 2.2.3. Microbiological Diagnosis 

Spontaneous or induced sputum samples were obtained from all the patients. Sputum samples were analyzed in the microbiology laboratory. Conventional semi-qualitative bacterial and fungal cultures were performed. An initial Gram staining was performed in all the samples prior to culturing the sputum if the Murray and Washington criteria were met [34] (Table S1). 

### 2.3. Blood Samples

In all the study patients and control subjects, blood samples were obtained from the arm vein after an overnight fasting period. Blood specimens were centrifuged at 1500 g for 15 min to collect the plasma samples and immediately frozen at −80 °C until further analyses.

### 2.4. Quantification of Oxidative Stress Markers and Cytokines 

*Oxidatively damaged DNA*. Levels of oxidative DNA adduct 8-hydroxy-2-deoxy guanosine (8-OHdG) were measured in plasma using the DNA Damage (8-OHdG) ELISA kit (StressMarq Biosciences INC., Victoria, BC, Canada) following the specific manufacturer’s instructions and previously described methodologies [8,35]. 

*Malondialdehyde (MDA)–protein adducts.* Levels of MDA–protein adducts were measured in plasma using the OxiSelect^TM^ MDA Adduct Competitive ELISA Kit (Cell Biolabs, Inc., San Diego, CA, USA) following the specific manufacturer’s instructions and previously described methodologies [8,35]. 

*Reduced glutathione (GSH)*. GSH was measured in the blood using the Human Reduced Glutathione (GSH) ELISA Kit (MyBioSource, San Diego, CA, USA) following the specific manufacturer’s instructions and previously described methodologies [8,35]. 

*Plasma levels of Trolox Equivalent Antioxidant Capacity (TEAC)*. TEAC levels were determined using the OxiSelect^TM^ Trolox Equivalent Antioxidant Capacity (TEAC Assay Kit (ABTS, Cell Biolabs, Inc., San Diego, CA, USA) following the manufacturer’s instructions. Twenty-five-microliter samples were added to the microplate well, and upon addition of 150 microL of the diluted 2,2′-azino-bis (3-ethylbenzothiazoline-6-sulfonic acid) reagent, samples were mixed thoroughly. Samples were then incubated on an orbital shaker for five minutes. Finally, the absorbance was read at 405 nm in all the sample wells. Antioxidant activity was determined by comparison with the Trolox standards. Intra-assay coefficients of variation for all the samples ranged from 0.39% to 9.96%. (Cell Biolabs, Inc., San Diego, CA, USA). The minimum detectable concentration of TEAC in plasma was set to be 250.29 g/mol (Cell Biolabs, Inc., San Diego, CA, USA).

*Quantification of myeloperoxidase in plasma.* Plasma levels of myeloperoxidase were measured using the Human Myeloperoxidase ELISA Kit (MyBioSource, San Diego, CA, USA) following the specific manufacturer’s instructions and previously described methodologies [8,35]. 

*Quantification of cyclooxygenase in plasma*. The Human Cyclooxygenase 2 ELISA Kit (MyBioSource, San Diego, CA, USA) was used following the specific manufacturer’s instructions and previously described methodologies [8,35]. 

### 2.5. Statistical Analysis

Normality of the study variables was tested using the Shapiro–Wilk test. Data are expressed as mean and standard deviation (SD) in tables and figures. The variables MDA-protein adducts, fibrinogen, and ceruloplasmin were used to calculate sample size. A minimum number of 23 patients and control subjects was required to achieve an 85% statistical power for those variables. Statistical significance was established at *p* ≤ 0.05. Potential differences of quantitative variables between the two groups were explored using the Student’s *t* test and Mann–Whitney U test (parametric and non-parametric distribution of the variables, respectively). Chi-square test was employed to assess potential differences in categorical variables (smoking history) between the two groups. Potential correlations between clinical, radiological, and biological variables were explored using the Pearson’s or the Spearman’s correlation coefficients. A Bonferroni-type adjustment was performed to considering the effect of having multiple correlations. In the post hoc analyses, in which the patients were subdivided on the basis of smoking history or pseudomona colonization, Kruskal–Wallis or analysis of variance (ANOVA) tests were used. The software Statistical Package for the Social Sciences (SPSS) 23.0 (SPSS Inc, Chicago, IL, USA) was used for this group of analyses. Correlations are displayed in graphical correlation matrixes, obtained from R package corrplot (https://cran.r-project.org/web/packages/corrplot/index.html, accessed on 21 May 2021), in different colors: blue for positive correlations and red for negative ones. Furthermore, comparisons among groups were also made on the basis of the degree of the disease severity according to the different scores (FACED, EFACED, and BSI), in which the percentages of patients in each category were depicted in histograms using MedCalc statistical software (Penn State University, World Campus, State College, Pennsylvania, PA, USA). 

## 3. Results

### 3.1. General Clinical Characteristics

Demographic variables are shown in Table 1. The variables age and sex did not significantly differ between the study groups. All the healthy controls were non-smokers. Patients exhibited mild airway obstruction compared to the controls. Lung volumes and diffusion capacity were preserved in bronchiectasis patients. Exercise capacity as measured by the walking test was significantly reduced in the patients compared to the controls. In the patients, radiological extension is also summarized in Table 1. 

Nutritional status as determined by BMI and FFMI and blood parameters were within normal ranges for both patients and controls, although albumin and prealbumin were significantly reduced in the patients compared to healthy subjects (Table 2). 

**Figure 1:**
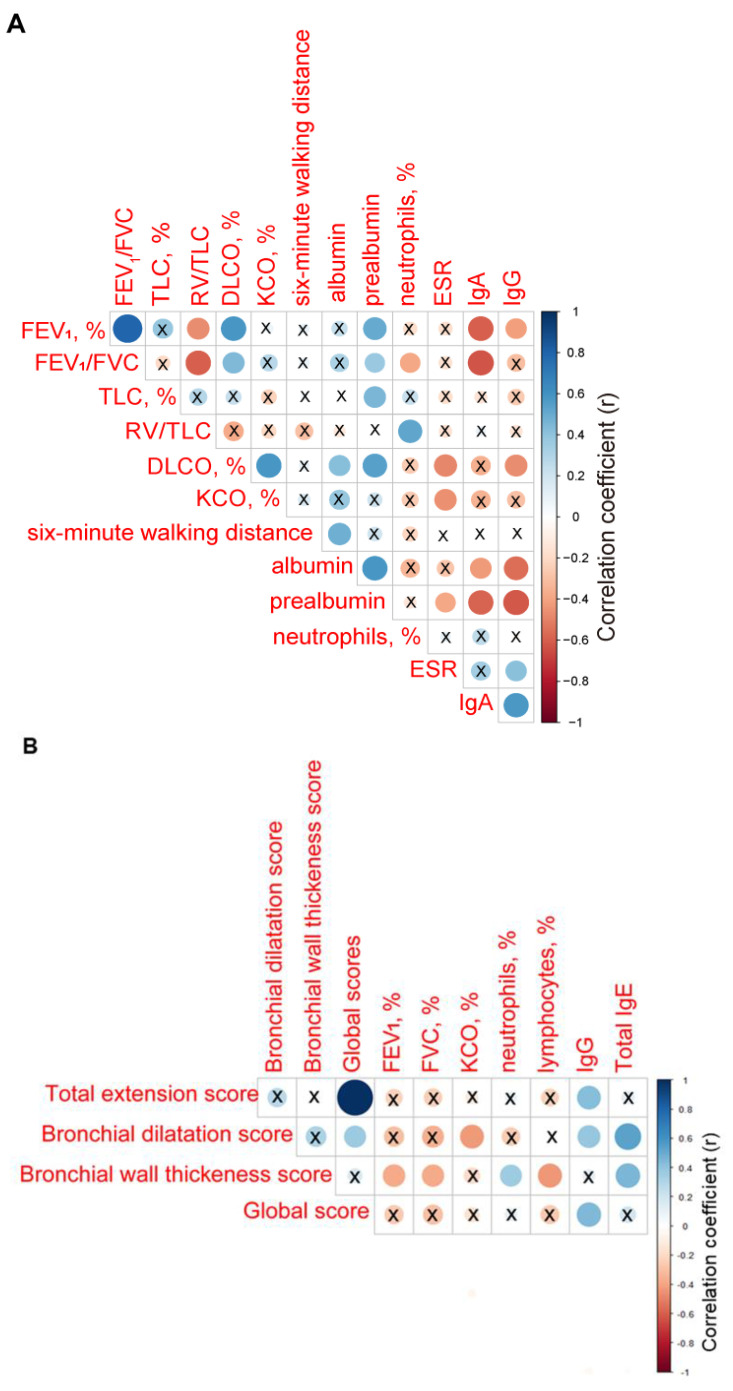
(**A**) Correlation matrix of the clinical and analytical variables. (**B**) Correlation matrix of radiological extension scores and clinical and analytical variables. In both matrices, positive correlations are represented in blue, while negative correlations are represented in red. The intersection within the circle represents *p* value > 0.05. Color intensity and the size of the circle are proportional to the correlation coefficients, as indicated in the *Y* axis on the right-hand side of the graph.

Among patients with bronchiectasis, the parameters FEV_1_, FEV1/FVC, TLC, and DL_CO_ significantly correlated with blood levels of prealbumin (r = 0.505, *p* = 0.004, r = 0.375, *p* = 0.041, r = 0.451, *p* = 0.021, and r = 0.550, *p* = 0.004, respectively, Figure 1A). Additionally, among the same patients, blood levels of albumin also correlated with DL_CO_ and six-minute walk distance (r = 0.426, *p* = 0.030 and r = 0.484, *p* = 0.008, respectively, Figure 1A). Inverse significant correlations were found between bronchial wall thickness scores and the lung function parameters FEV_1_ and FVC (r = −0.379, *p* = 0.039 and r = −0.371, *p* = 0.044, respectively, Figure 1B), and lymphocyte counts (r = −0.433, *p* = 0.017, Figure 1B), and a positive correlation with neutrophil counts (r = 0.360, *p* = 0.050, Figure 1B). Total global scores positively correlated with immunoglobulin G (IgG) levels (r = 0.447, *p* = 0.013, Figure 1B). 

### 3.2. Disease Severity

**Figure 2:**
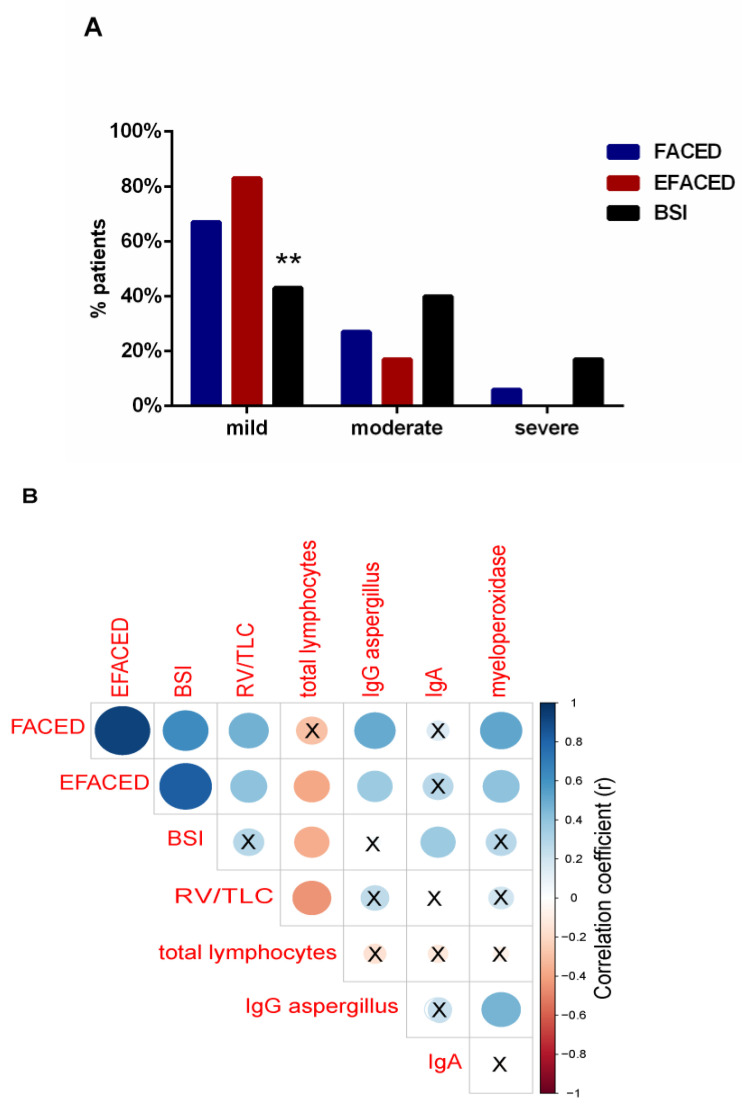
(**A**) Histograms of the proportions of patients according to disease severity: mild, moderate, and severe, according to FACED (blue histograms), EFACED (red histograms), and BSI (black histograms). Statistical significance: ** *p* ≤ 0.01 between BSI and EFACED proportions of patients. (**B**) Correlation matrix of the disease severity scores and analytical variables, in which positive correlations are represented in blue, while negative correlations are represented in red. The intersection within the circle represents *p* value > 0.05. Color intensity and the size of the circle are proportional to the correlation coefficients, as indicated in the *Y* axis on the right-hand side of the graph.

The use of the severity scores FACED, EFACED, and BSI revealed that the majority of the patients had mild disease (67%, 83% and 43%, respectively, Figure 2A). EFACED and BSI scores negatively correlated with total lymphocytes (r = −0.384, *p* = 0.360 and r = −0.366, *p* = 0.047, Figure 2B). Moreover, BSI score positively correlated with immunoglobulin A (IgA) levels (r = 0.365, *p* = 0.047, Figure 2B). Both FACED and EFACED scores positively correlated with myeloperoxidase (r = 0.530, *p* = 0.003 and r = 0.403, *p* = 0.027, respectively, Figure 2B).

### 3.3. Etiology and Microbiology of Bronchiectasis Patients

Most of the patients had post-infectious bronchiectasis, including tuberculosis (Table 3). 

Moreover, the presence of microorganisms in the sputum of all the patients is listed in Table 4. Approximately one third of the patients had *Pseudomona aeruginosa* in their sputum samples (Table 4). 

### 3.4. Systemic Oxidative Stress Markers

**Figure 3:**
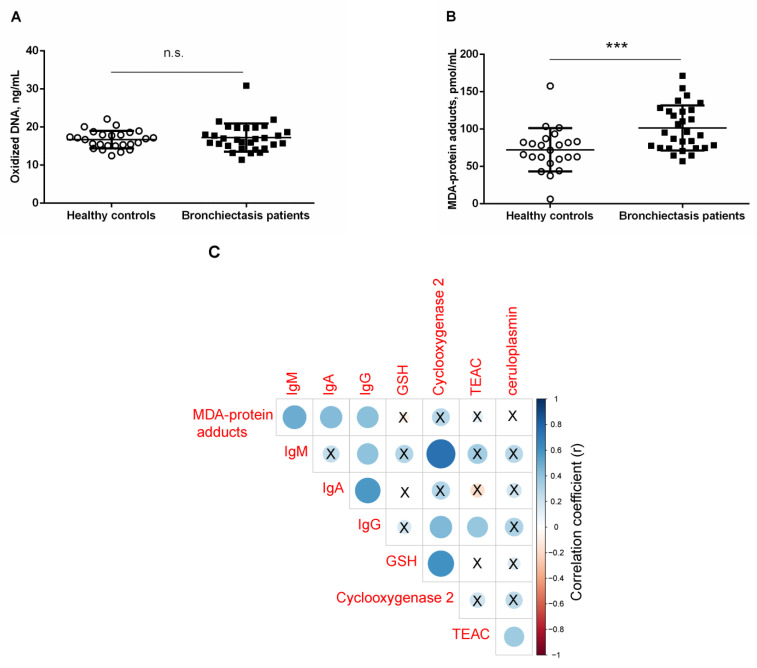
Mean values and standard deviations of plasma (**A**) oxidized DNA levels (ng/mL) and (**B**) MDA–protein adducts (pmol/mL) in healthy controls and bronchiectasis patients. Statistical significance: *** *p* ≤ 0.001 between bronchiectasis patients and healthy controls; and n.s., non-significant differences between the two study groups. (**C**) Correlation matrix of the disease among different biological and analytical variables, in which positive correlations are represented in blue, while negative correlations are represented in red. The intersection within the circle represents *p* value > 0.05. Color intensity and the size of the circle are proportional to the correlation coefficients, as indicated in the *Y* axis on the right-hand side of the graph.

*Prooxidants*. Levels of oxidized DNA did not significantly differ between bronchiectasis patients and healthy controls, even when patients were subdivided according to smoking history or pseudomona colonization (Figure 3A and Tables S1 and S2). Plasma MDA–protein adduct levels were significantly higher in bronchiectasis patients, independently of smoking history or pseudomona infection, compared to control subjects (Figure 3B and Tables S1 and S2). In bronchiectasis patients, positive significant correlations were detected between MDA–protein adduct levels and immunoglobulin M (IgM), IgA, and IgG levels (r = 0.499, *p* = 0.006, r = 0.438, *p* = 0.017, and r = 0.416, *p* = 0.025, respectively, Figure 3C).

**Figure 4:**
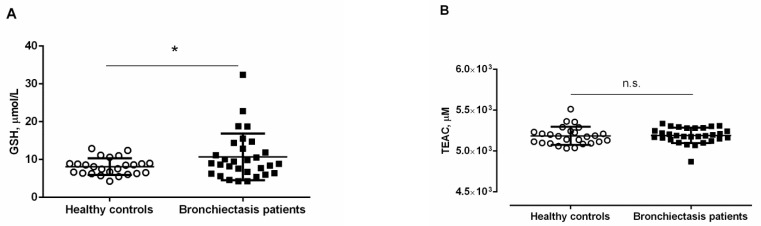
Mean values and standard deviations of plasma (**A**) GSH levels (µmol/L) and (**B**) TEAC levels (µM) in healthy controls and bronchiectasis patients. Statistical significance: * *p* ≤ 0.05 between bronchiectasis patients and healthy controls; and n.s., non-significant differences between the two study groups.

*Antioxidants.* Plasma GSH levels were significantly greater in bronchiectasis patients, especially in the ex-smokers and in the pseudomona-colonized patients, than in healthy controls (Figure 4A and Tables S2 and S3). Furthermore, plasma GSH levels were significantly higher in ex-smokers and pseudomona-colonized patients than in non-smokers and non-pseudomona patients, respectively (Tables S2 and S3). 

No significant differences were detected in plasma TEAC levels between the two study groups, and smoking history or pseudomona colonization did not influence the results (Figure 4B and Tables S2 and S3). Significant positive associations were detected between plasma levels of GSH and cyclooxygenase-2 (r = 0.607, *p* < 0.001, Figure 3C). Significant positive associations were also observed between TEAC levels and either IgG or ceruloplasmin (r = 0.384, *p* = 0.036 and r = 0.358, *p* = 0.050, respectively, Figure 3C).

### 3.5. Systemic Inflammatory Markers

The percentage of blood neutrophils was significantly greater in the patients compared to healthy controls (Table 5 and Tables S2 and S3). Conversely, total blood lymphocyte counts and the percentage of these cells were significantly lower in the patients than in the healthy controls (Table 5 and Tables S2 and S3). Importantly, levels of the plasma inflammatory parameters c-reactive protein (CRP), erythrocyte sedimentation rate (ESR), fibrinogen, alpha-1 antitrypsin, and ceruloplasmin were significantly higher in bronchiectasis patients, irrespective of smoking history, and particularly non-pseudomona infected patients compared to healthy controls (Table 5 and Table S2). Moreover, plasma levels of IgA and IgG were significantly greater in bronchiectasis patients, particularly in those infected by pseudomona, than in healthy controls (Table 5 and Tables S2 and S3). 

**Figure 5:**
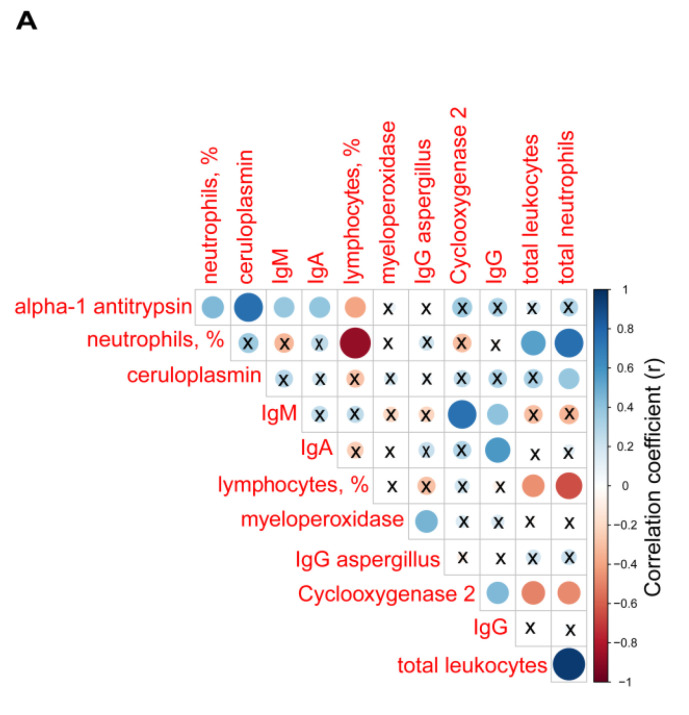

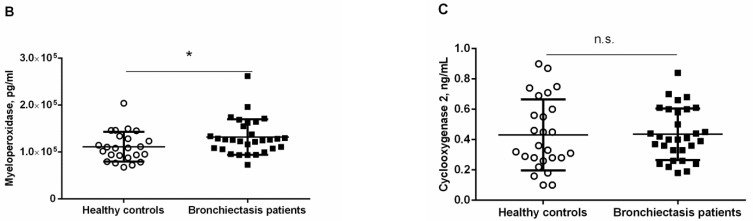
(**A**) Correlation matrix of biological and analytical variables, in which positive correlations are represented in blue, while negative correlations are represented in red. The intersection within the circle represents *p* value > 0.05. Color intensity and the size of the circle are proportional to the correlation coefficients, as indicated in the *Y* axis on the right-hand side of the graph. Mean values and standard deviations of plasma (**B**) myeloperoxidase levels (pg/mL) and (**C**) cyclooxygenase levels (ng/mL) in healthy controls and bronchiectasis patients. Statistical significance: * *p* ≤ 0.05 between bronchiectasis patients and control subjects and n.s., non-significant differences between the study groups.

Among the bronchiectasis patients, positive significant correlations were detected between alpha-1 antitrypsin levels and the percentage of neutrophils, ceruloplasmin, IgM, and IgA (r = 0.441, *p* = 0.015, r = 0.758, *p* < 0.001, r = 0.381, *p* = 0.038, and r = 0.396, *p* = 0.030, respectively, Figure 5A), while a negative correlation was seen between alpha-1 antitrypsin levels and the percentage of lymphocytes (r = −0.393, *p* = 0.032, Figure 5A). 

Plasma levels of myeloperoxidase were significantly higher in bronchiectasis patients than in healthy controls (Figure 5B and Table S2). Pseudomona colonization did not significantly influence plasma myeloperoxidase levels among the patients (Table S3). Plasma levels of cyclooxygenase-2 did not significantly differ between bronchiectasis patients and the controls (Figure 5C). However, plasma levels of clyclooxygenase were greater in the ex-smokers than in the non-smoker patients (Table S2). 

Plasma levels of myeloperoxidase positively correlated with IgG aspergillus levels (r = 0.460, *p* = 0.010, Figure 5A). Plasma levels of cyclooxygenase-2 also positively correlated with IgM and IgG (r = 0.741, *p* < 0.001 and r = 0.444, *p* = 0.014, Figure 5A), whereas inverse correlations were observed between cyclooxygenase-2 levels and either total leukocyte or neutrophil counts (r = −0.498, *p* = 0.005 and r = −0.474, *p* = 0.008, respectively, Figure 5A).

## 4. Discussion

In the current investigation, the most relevant findings were that in stable bronchiectasis patients with relatively mild disease, levels of parameters indicative of systemic inflammation and oxidative stress were significantly increased compared to a population of healthy subjects. Patients exhibited very mild airway obstruction, preserved lung volumes and diffusion capacity, and exercise tolerance was reduced, although within normal ranges, compared to the healthy controls. Acute phase reactant levels along with neutrophil percentages were increased in the stable patients compared to control subjects. These relevant findings are discussed below. 

In the current investigation, patients had mild–very mild disease, as demonstrated by the disease severity scores and lung function parameters. The majority of the patients were classified as mild according to BSI and both FACED and EFACED scores, particularly the latter ones. In fact, greater proportions of patients fell into the category of mild disease when using the FACED and EFACED scores than with the BSI classification. Differences between the two types of scales are attributable to the fact that BSI contains more items for each variable [25,26]. Furthermore, patients exhibited mild airway obstruction, and exercise capacity as measured by walking distance was also mildly reduced compared to the control subjects. Nutritional parameters were also within the normal range in this cohort of patients with post-infectious bronchiectasis in most of the cases. These results confirm that the study population met the inclusion criteria for the purpose of the investigation.

Acute exacerbations are associated with increased levels of several systemic inflammatory molecules, namely proinflammatory cytokines, especially in patients with infection by *Pseudomona aeruginosa* [36]. Systemic inflammatory parameters, particularly tumor necrosis factor (TNF)-alpha, fibrinogen, ESR, and CRP, were also significantly higher in patients with mild-to-moderate bronchiectasis compared to healthy controls [19]. Moreover, high plasma levels of TNF-alpha were associated with worse disease severity, as identified by greater computed tomography (CT) scan scores, higher airway obstruction, and colonization by *Pseudomona aeruginosa* [19]. Importantly, in the present study, acute phase reactants (CRP, ESR, fibrinogen, alpha-1 antitrypsin, and ceruloplasmin), neutrophil counts, and myeloperoxidase levels were all significantly increased in this cohort of patients with stable mild bronchiectasis, in whom the prevalence of *Pseudomona aeruginosa* colonization was low (only one third of the patients). Besides, diffusion capacity was negatively associated with ESR among the patients. Importantly, patients with no colonization by *Pseudomona aeruginosa* were those with a significant rise in plasma CRP, ESR, fibrinogen, and ceruloplasmin compared to the controls. In non-smoker patients, levels of ESR and fibrinogen also significantly increased compared to controls. Thus, smoking history did not influence these results. Collectively, these findings suggest that systemic inflammation is very prominent in bronchiectasis, even in patients with very stable disease (no acute exacerbations in the previous 6–12 months for some of the patients) showing no nutritional abnormalities, active airway infections, or any other systemic manifestations. As far as we are concerned, these are relevant novel findings as patients in previous series exhibited more severe disease and/or reported a history of microbial colonization [19,36]. It should also be mentioned that cyclooxygenase plasma levels did not differ between patients as a whole and healthy controls, implying that this pathway was not involved in the pathophysiology of systemic inflammation associated with bronchiectasis. Nonetheless, a rise in plasma cyclooxygenase levels was detected in the ex-smokers compared to non-smoker patients. These results imply that cigarette smoke exposure may induce cyclooxygenase, as previously stated [37]. Plasma levels of myeloperoxidase were, indeed, higher in the patients than in the controls. As bronchiectasis is a rather neutrophilic disease, as also confirmed in our study, levels of the enzyme myeloperoxidase were expectedly increased in the patients. 

In the present study, plasma levels of IgA and IgG were significantly increased in the patients compared to the controls. Patients with colonization by pseudomona were those exhibiting the greatest levels of both IgA and IgG. These results imply that none of the patients had immunodeficiencies in this series, as previously reported in other cohorts [38,39]. Conversely, in another series, high levels of IgA and IgG were inversely correlated with lung function deterioration and disease extension (CT scans) [9]. In that study, however, a control group of patients was not included in the investigation. In the present investigation, levels of IgG and IgA were significantly greater in the patients than in the group of healthy controls. Moreover, inverse correlations were also detected between either IgA or IgG and the degree of airway obstruction (FEV_1_, FEV_1_/FVC) and diffusion capacity (DL_CO_). A significant positive correlation was also detected between IgG levels and total global scores of radiological extension. Despite the fact that patients exhibited mild disease (low disease severity scores and preserved lung function), radiological extension may account for the increased levels of all the acute phase reactants and immunoglobulins. Interestingly, inverse correlations were also found between the degree of airway obstruction of the patients and the radiological parameter bronchial wall thickness, as also previously demonstrated in another investigation [40].

Oxidants that escape the antioxidant systems target several cellular structures, among which proteins may be severely affected. Reactive carbonyl derivatives (aldehydes and ketones) result from the reaction of oxidants with several amino acids in the protein side-chains. In addition, Michael addition reactions of other amino acid residues with α,β-unsaturated aldehydes resulting from the peroxidation of polyunsaturated fatty acids also lead to the formation of reactive carbonyls, which can be detected in tissues using specific antibodies [41,42,43]. Increased levels of MDA–protein adducts have been shown in several compartments of patients with COPD and in lung cancer [11,13,31,44]. In the present study, a rise in plasma MDA–protein adducts was also detected for the first time among patients with stable bronchiectasis. Similarly, an increase in reactive carbonyls and superoxide anion levels was also reported in a cohort of bronchiectasis patients with moderate-to-severe airway obstruction [10]. In this study, however, disease severity was not assessed in the study patients. Significant correlations were observed between plasma MDA–protein adduct levels and IgG, IgM, and IgA, suggesting that disease extension may be associated with levels of oxidative stress. 

Cells contain several enzymatic and non-enzymatic antioxidants to fight against the deleterious effects of oxidants. Superoxide dismutase isoforms, catalase, and glutathione peroxidases are abundantly expressed antioxidant enzymes. The action of antioxidant enzymes is complemented by the effects of non-enzymatic antioxidant systems. As such, glutathione, which is a water-soluble compound, is extensively distributed in organs and tissues. Reduced glutathione (GSH) levels are indicative of the redox potential of a tissue. In children with CF (3 years of age), low levels of GSH due to an inherent glutathione deficiency detected in the airways occurred early in life, which enhanced the oxidative stress response during infections [45]. 

In the current investigation, however, plasma levels of GSH were increased in the stable non-CF bronchiectasis patients. Such an increase was particularly evident among patients colonized by *Pseudomona aeruginosa* and the ex-smokers. These findings suggest that the rise in GSH was most likely trying to counterbalance the deleterious effects of increased plasma protein oxidation levels, especially among patients with *Pseudomona aeruginosa* colonization. Other markers of oxidative stress also analyzed in the study (oxidized DNA and TEAC) did not seem to play any significant role in this cohort of patients as no significant differences from the healthy controls were observed. 

### Study Limitations

In the current investigation, a greater number of patients might have been recruited for the purpose of the study. However, sample size calculation showed that a sample of 23 patients was sufficient to reach 85% statistical power. Moreover, the investigation focused mainly on the assessment of patients with mild and very mild disease, in whom a significant rise in plasma inflammatory and oxidative stress balance was already observed. Other series, however, have reported significant changes in patients with more advanced and larger extension bronchiectasis [10,45]. Furthermore, future investigations with a larger cohort of patients will have to address further questions such as whether the profile of bronchial infection including those with no bacterial predominance (normal flora) may determine variations in the systemic patterns of inflammation and redox balance in patients with mild bronchiectasis. Additionally, future research should be devoted to the study of the mechanisms whereby increased oxidative stress and inflammation may precede other systemic manifestations, namely nutritional and muscle status alterations.

## 5. Conclusions

In stable patients with mild bronchiectasis, several important inflammatory and oxidative stress events take place in plasma. These findings suggest that the extension of bronchiectasis probably plays a role in the development of redox imbalance and inflammation in patients with mild bronchiectasis. Increased inflammation and oxidative stress in bronchiectasis may precipitate the occurrence of additional systemic manifestations in these patients, especially those related to muscle and nutritional abnormalities. These findings have therapeutic implications in the management of bronchiectasis patients.

## Figures and Tables

**Table 1:** Clinical characteristics in bronchiectasis patients and healthy controls.

	Healthy Controls	Bronchiectasis Patients
	N = 26	N = 30
**Clinical characteristics**, mean (SD)		
Age, years	61 (11)	66 (12)
Female, N/male, N	17/9	23/7
**Smoking history**		
Ex-smokers, N (%)	0	9 (30) **
Never smokers, N (%)	26 (100)	21 (70) **
Packs-year, mean (SD)	NA	22 (15)
**Lung functional assessment**, mean (SD)		
FEV_1_, % predicted	100 (13)	76 (25) ***
FVC, % predicted	100 (12)	85 (18) ***
FEV_1_/FVC	80 (9)	68 (11) ***
RV, % predicted	NA	151 (36)
TLC, % predicted	NA	102 (16)
RV/TLC	NA	55 (9)
DL_CO_, % predicted	NA	76 (15)
K_CO_, % predicted	NA	80 (11)
**Exercise capacity**, mean (SD)		
6-min walking distance, meters	536 (72)	473 (96) *
Distance, % predicted	107 (12)	98 (17) *
**Disease severity**		
FACED score, mean (SD)	NA	1.9 (1.3)
Mild, N	NA	20
Moderate, N	NA	8
Severe, N	NA	2
EFACED score, mean (SD)	NA	2.1 (1.5)
Mild, N	NA	25
Moderate, N	NA	5
Severe, N	NA	0
BSI score, mean (SD)	NA	5.5 (3.2)
Mild, N	NA	13
Moderate, N	NA	12
Severe, N	NA	5
**Radiological extension**, mean (SD)		
Total extension score	NA	8.1 (3.3)
Bronchial dilatation score	NA	1.2 (0.2)
Bronchial wall thickness score	NA	1.3 (0.3)
Global score	NA	10.6 (3.4)

Continuous variables are presented as mean (standard deviation), while categorical variables are presented as the number of patients in each group along with the percentage for the study group. Abbreviations: N, number; NA, not applicable; FEV_1_, forced expiratory volume in the first second; FVC, forced vital capacity; RV, residual volume; TLC, total lung capacity; DL_CO_, carbon monoxide transfer; K_CO_, Krogh transfer factor. FACED: F, FEV_1,_ force expiratory volume in the first second; A, Age; C, chronic colonization by Pseudomonas aeruginosa; E, radiological extension; D, dyspnea; EFACED: E, exacerbations with hospitalization in previous year; F, FEV_1,_ force expiratory volume in the first second; A, Age; C, Chronic colonization by Pseudomonas aeruginosa; E, radiological extension; D, dyspnea; BSI, Bronchiectasis Severity Index. FACED: mild: 0–2, moderate: 3–4, severe: 5–7; EFACED: mild: 0–3, moderate: 4–6, severe: 7–9; BSI: mild: 0–4, moderate: 5–8, severe: ≥ 9. Statistical analyses and significance: *, *p* ≤ 0.05; **, *p* ≤ 0.01; ***, *p* ≤ 0.001 between bronchiectasis patients and control subjects.

**Table 2:** Nutritional assessment in bronchiectasis patients and healthy controls.

	Healthy Controls	Bronchiectasis Patients
	N = 26	N = 30
**Nutritional parameters**, mean (SD)		
BMI (kg/m^2^)	27 (4)	25 (4)
FFMI (kg/m^2^)	17 (2)	16 (3)
Hemoglobin, g/dL	14.3 (1.3)	13.9 (1.1)
Hematocrit, %	42.9 (3.9)	42.0 (3.7)
Glucose, mg/dL	102.9 (20.6)	94.4 (25.5)
Creatinine, mg/dL	0.8 (0.2)	0.7 (0.3)
Albumin, g/dL	4.6 (0.2)	4.4 (0.3) ***
Total proteins, g/dL	7.3 (0.3)	7.3 (0.4)
Prealbumin, g/dL	26.0 (4.9)	22.1 (5.0) **

Values are presented as mean (standard deviation). Abbreviations: N, number; BMI, body mass index; kg, kilograms; m, meters; FFMI, fat-free mass index; g, grams; dL, deciliter; mg, milligrams. Statistical analyses and significance: **, *p* ≤ 0.01; ***, *p* ≤ 0.001 between bronchiectasis patients and control subjects.

**Table 3:** Etiology of bronchiectasis in the study patients.

	Bronchiectasis Patients
	N = 30
**Etiology**	
Post-infectious, N (%)	22 (73)
COPD, N (%)	1 (3)
Unknown etiology, N (%)	7 (24)

Abbreviations: N, number; COPD, Chronic Obstructive Pulmonary Disease.

**Table 4:** Microbiological status of the study patients.

Patients	Germs	Score
Patient # 1	Haemophilus influenza, S	5
Patient # 2	Moraxella catarrhalis, S	5
Patient # 3	Pseudomona aeruginosa, S	3
Patient # 4	Pseudomona aeruginosa, S	3
Patient # 5	Commensal microbiota, S	5
Patient # 6	Pseudomona aeruginosa, S	5
Patient # 7	Commensal microbiota, S	6
Patient # 8	Pseudomona aeruginosa, S	5
Patient # 9	Pseudomona aeruginosa, S	5
Patient # 10	Commensal microbiota, S	5
Patient # 11	Commensal microbiota, S	5
Patient # 12	Commensal microbiota, S	5
Patient # 13	NSA, I	NA
Patient # 14	Pseudomona aeruginosa, S	5
Patient # 15	Commensal microbiota, S	6
Patient # 16	Pseudomona aeruginosa, S	5
Patient # 17	Commensal microbiota, S	5
Patient # 18	NSA, I	NA
Patient # 19	Commensal microbiota, S	5
Patient # 20	Commensal microbiota, S	5
Patient # 21	Commensal microbiota, S	6
Patient # 22	NC, S	2
Patient # 23	Commensal microbiota, S	3
Patient # 24	NSA, I	NA
Patient # 25	NSA, I	NA
Patient # 26	Commensal microbiota, S	6
Patient # 27	Pseudomona aeruginosa, S	6
Patient # 28	Commensal microbiota, S	6
Patient # 29	Pseudomona aeruginosa, S	6
Patient # 30	Commensal microbiota, S	6

Abbreviations: S, spontaneous; I, induced; NSA, no sputum available; NA, not available; NC, no culture.

**Table 5:** Systemic inflammatory parameters in bronchiectasis patients and healthy controls.

	Healthy Controls	Bronchiectasis Patients
	N = 26	N = 30
**Systemic inflammatory parameters**, mean (SD)		
Total leukocytes, ×10^3^/µL	6.3 (1.6)	6.4 (1.6)
Total neutrophils, ×10^3^/µL	3.9 (1.2)	4.1 (1.4)
Neutrophils, %	57.6 (6.9)	63.2 (7.8) **
Total lymphocytes, ×10^3^/µL	2.1 (0.6)	1.5 (0.40) ***
Lymphocytes, %	31.7 (6.3)	24.5 (6.2) ***
Total eosinophils, ×10^3^/ µL	0.15 (0.12)	0.16 (0.09)
Eosinophils, %	2.3 (1.6)	2.5 (1.4)
Platelets, ×10^3^/µL	246 (63)	257 (69)
CRP, mg/dL	0.23 (0.4)	0.70 (0.9) *
ESR, mm/h	8 (7)	15 (12) **
Fibrinogen, mg/dL	305 (69)	370 (84) **
Alpha-1 antitrypsin, mg/dL	117.9 (18.3)	132.5 (25.4) *
Ceruloplasmin, mg/dL	22.7 (5.1)	27.0 (5.4) **
IgE, IU/mL	44 (42)	66 (81)
IgG aspergillus, mg/L	26 (23)	37 (35)
IgM, mg/dL	96 (44)	112 (85)
IgA, mg/dL	249 (131)	330 (134) *
IgG, mg/dL	1089 (199)	1273 (384) *

Values are presented as mean (standard deviation). Abbreviations: N, number; CRP, C-reactive protein; ESR, erythrocyte sedimentation rate; IgE, immunoglobulin E; IgG aspergillus, immunoglobulin G aspergillus; IgM, immunoglobulin M; IgA, immunoglobulin A; IgG, immunoglobulin G; µL, microliter; mg, milligrams; mm, millimeters; h, hour; dL, deciliter; IU, international unit; dL, deciliter. Statistical analyses and significance: *, *p* ≤ 0.05; **, *p* ≤ 0.01; ***, *p* ≤ 0.001 between bronchiectasis patients and control subjects.

## Data Availability

The datasets are available from the corresponding authors upon reasonable request.

## Supplementary Materials

The following are available online at https://www.mdpi.com/article/10.3390/jcm10194534/s1, Table S1: Criteria for evaluation of the quality of sputum specimens, Table S2: Systemic inflammatory and oxidative stress parameters in bronchiectasis patients according to smoking history, Table S3: Systemic inflammatory and oxidative stress parameters in bronchiectasis patients according to pseudomona aeruginosa colonization.
