# Supplementary material for: Do Redox Balance and Inflammatory Events Take Place in Mild Bronchiectasis? A Hint to Clinical Implications
Source: J Clin Med. 2021 Sep 30;10(19):4534. doi: 10.3390/jcm10194534 (PMC8509750; doi:10.3390/jcm10194534)
Supplement: Supplementary file 1 [file jcm-10-04534-s001.zip › jcm-1314093-supplementary.pdf]

Supplementary material

Do Redox Balance and Inflammatory Events Take Place in Mild Bronchiectasis? A Hint to Clinical Implications

[1]Liyun Qin, [1,2]Maria Guitart, [1,2]Mireia Admetlló, [3]Sandra Esteban-Cucó, [4]José María Maiques, [1,5]Yingchen Xia, [1,5]Jianhua Zha, [4]Santiago Carbullanca, [6]Xavier Duran, [1]Xuejie Wang, [1,2]Esther Barreiro

METHODS

Study subjects

This was a prospective, controlled, cross-sectional study, in which 30 patients (7 males) were recruited consecutively from the Multidisciplinary Bronchiectasis Unit of the Respiratory Department at Hospital del Mar (Barcelona, Spain) over the years 2019-2020. Additionally, 26 age- and sex-matched control subjects (9 males) were recruited from the general population (patients' relatives or friends) at Hospital del Mar. In the patients, inclusion criteria were as follows: adults (18 years and over), diagnosis of non-CF bronchiectasis by high-resolution computerized tomography (HRCT) [1,2], no previous exacerbations of the disease at least 4 weeks prior to study entry (range from 2 months to four years). Exclusion criteria for all the patients and control subjects included other chronic cardiovascular or respiratory disorders, chronic metabolic diseases, signs of severe bronchial inflammation and/or infection, current or recent invasive mechanical ventilation, long-term oxygen therapy, and poor collaboration. Most of the patients recruited for the purpose of the investigation had a mild-to-moderate disease severity on the basis of lung function impairment, disease scoring using several indices, and radiologic extension [1,3–6]. All the

patients were stable at the time of study entry. Approval was obtained from the institutional Ethics Committee on Human Investigation (Hospital del Mar-IMIM, Barcelona, protocol # 2019/8482/1) following the World Medical Association guidelines (Declaration of Helsinki, Fortaleza, Brazil, October 2013) for research on human beings. Informed written consent was obtained from all participants.

Clinical Assessment

Nutritional evaluation included the assessment of body mass index (BMI) and determination of fat-free mass index (FFMI) using bioelectrical impedance [7–11]. Blood analytical parameters (systemic inflammatory cells and markers) were also obtained in all participants. Lung function parameters were determined in all study subjects following standard procedures [7–11]. Exercise capacity was assessed through the 6-minute walking distance following previous methodologies [12]. Etiology of the non-CF bronchiectasis of all the study patients was also assessed.

Bronchiectasis severity scores

The FACED score [3] was used for clinical estimation of the patients' status by incorporating variables: forced expiratory volume in 1 s (FEV_1) [F; cutoff, 50%; 0 or 2 points], age [A; cutoff, 70 years; 0 or 2 points], chronic colonization by *Pseudomonas aeruginosa* [C; yes, 0 or 1 point], radiological extension [E; number of lobes affected; cutoff, two lobes; 0 or 1 point], and dyspnea [D; cutoff, grade 2 on the Medical Research Council dyspnea scale; 0 or 1 point]). Based on the total score, bronchiectasis is evaluated into three groups (mild, 0–2 points; moderate, 3–4 points; and severe, 5-7 points).

The EFACED score [4] (E: exacerbations with hospitalization in previous year; F: FEV_1; A:

age; C: chronic colonization by *Pseudomonas aeruginosa*; E: radiological extension [number of pulmonary lobes affected]; and D: dyspnea). An EFACED score of 0-3 is mild, 4-6 is moderate and 7-9 is severe.

The BSI score [5] (age [maximum value: 6 points], BMI [maximum value: 2 points], FEV_1 [maximum value: 3 points], hospital admission prior to study [maximum value: 5 points], exacerbations prior to the study [maximum value: 2 points], MRC dyspnea scale [maximum value: 3 points], chronic colonization by *Pseudomonas aeruginosa* [maximum value: 3 points], chronic colonization by other microorganisms [maximum value: 1 points], radiological extension [maximum value: 1 points]). Bronchiectasis was evaluated in three groups according to the severity scores (mild, 0–4 points; moderate, 5-8 points; and severe, ≥ 9 points).

Radiological extension

High-resolution computer tomography (HRCT)-scans were used to evaluate the radiological extension of the bronchiectasis in all the study patients. Scores for each patient were calculated by two independent observers following previously established criteria [13,14]. The extent of bronchiectasis (ES) was scored for each lobe as follows: grade 0 = no disease; grade 1= one or partial bronchopulmonary segment involved; grade 2 = two or more bronchopulmonary segments involved. The lingula lobe was considered as an independent one in this analysis. The bronchial dilatation (DS) was quantified relative to the adjacent pulmonary arteries as follows: grade 0 = no bronchiectasis; grade 1 = less than twice (200%) diameter of adjacent pulmonary artery (APA); grade 2 = 200–300% diameter of APA; grade 3 = >300% diameter of APA. Bronchial wall thickness (TS) was scored as follows: grade 0 =

none; grade 1 = 50% of APA, grade 2 = 50–100% of APA; grade 3 = >100% of APA.

The CT scans were also scored for parenchymal items in the six lobes: collapse or consolidation, mucus plugging, emphysema and fibrosis or retraction. Collapse or consolidation: subsegmental collapse or consolidations = 1 and segmental or lobar collapse or consolidations = 2. Mucus plugging was scored as follows: the presence of subsegmental mucus plugging = 1 and in case of the presence of segmental or lobar mucus plugging = 2. Emphysema was recorded as follows: subsegmental emphysema =1 and segmental or lobar emphysema= 2. Fibrosis or retraction was scored as follows: subsegmental fibrosis or retraction = 1 and segmental or lobar fibrosis or retraction = 2.

Global scores of both lungs were taken for extension, bronchial dilatation and bronchial wall thickness. The total extent of bronchiectasis (TES) was taken as the sum of the ES for each of the six lobes. The global severity of bronchial dilatation (GDS) was estimated using a weighted average, calculated as the "sum of the extent score multiplied by the dilatation score for each lobe", divided by the "total extent score" ($GDS = \sum(ES*DS)_{1-6}/TES$). Similarly, the global severity of bronchial wall thickness (GWTS) was estimated as the "sum of the extent score multiplied by the thickness score for each lobe" divided by the "total extent score" ($GTS = \sum(ES*TS)_{1-6}/TES$).

Microbiological diagnosis

Spontaneous or induced sputum samples were obtained from all the patients. Sputum samples were analyzed in the microbiology laboratory. Conventional semi-qualitative bacterial and fungal cultures were performed. An initial Gram staining was performed in all the samples prior to culturing the sputum if the Murray and Washington criteria were

[15] (Table S1).

Table S1. Criteria for evaluation of the quality of sputum specimens

Score	Epithelial cells	Leukocytes	Quality	Culture
1	>25	<10	Very poor	No
2	>25	18-25	Poor	No
3	>25	>25	Dubious	Yes
4	18-25	>25	Sufficient	Yes
5	<10	>25	Good	Yes
6	<25	<25	Uncertain	Yes

Adapted from Murray P.R. et al. See reference # 15.

Sputum samples were cultured in Chocolate agar, Columbia Naladixic Acid (CNA) agar, MacConkey agar and Sabouraud Agar. Bacterial cultures were read at 24h and 48h time-points, while those of fungal cultures were read every 24h for five consecutive days. Antibiotic sensitivity was tested using the microdilution method or disc diffusion following the regulations of the European Committee on Antimicrobial Susceptibility Testing (EUCAST) [16]. These strains were frozen in two separate freezing tubes at -80ºC.

When available, mycobacteria cultures were also performed in the patients. Upon sample decontamination using the sodium hydroxide (NaOH) method, samples were cultured in solid medium culture of Lowenstein Jensen (BLL) and liquid medium BACT/ALERT® (BioMerieux, SA F-69280 Marcy I'Etoile, France) or BACTEC™ MGIT™ 960 (BD) for 40 consecutive days.

Blood samples

In all the study patients and control subjects, blood samples were obtained from the arm vein after an overnight fasting period. Blood specimens were centrifuged at 1,500 g for 15 minutes to collect the plasma samples and immediately frozen at -80ºC until further analyses.

Quantification of oxidative stress markers and cytokines

Oxidatively damaged DNA. Levels of oxidative DNA adduct 8-hydroxy-2-deoxy guanosine (8-OHdG) were measured in plasma using the DNA Damage (8-OHdG) ELISA kit (StressMarq Biosciences INC., Victoria, BC, Canada) following the specific manufacturer's instructions and previously described methodologies [17,18]. Briefly, 50 microL of plasma was incubated with 50 microL of antibody per well at room temperature for one hour in a plate cover. After several washes, samples were incubated with 3,3′,5,5′-tetramethylbenzidine (TMB) substrate in the dark at room temperature for 30 minutes. Immediately afterwards, 100 microL of stop solution was poured into each well. Samples were then shaken from side to side and thoroughly mixed with the solution. After terminating this reaction, the absorbance was read at 450 nm in all the sample wells. A standard curve was always generated with each assay run. Intra-assay coefficients for all the samples ranged from 0.11% to 9.62%. The minimum detectable concentration of DNA in plasma was set to be 0.94 ng/mL (StressMarq Biosciences INC.).

Malondialdehyde (MDA)-protein adducts. Levels of MDA-protein adducts were measured in plasma using the OxiSelect™ MDA Adduct Competitive ELISA Kit (Cell Biolabs, Inc., San Diego, CA, USA) following the specific manufacturer's instructions and previously

described methodologies [17,18]. First, an MDA conjugate was coated on an ELISA plate, then 50 microL of plasma specimens were added to the MDA conjugate preabsorbed ELISA plate and incubated at room temperature for 10 minutes on an orbital shaker. After a brief incubation, the primary antibody was added and incubated at room temperature for one hour on an orbital shaker. After three washes, samples were incubated with secondary antibody at room temperature for another hour on an orbital shaker. After three washes, the substrate solution was added at room temperature for 20 minutes and samples were again shaken on an orbital shaker. Immediately afterwards, 100 microL of the stop solution was poured into each well. Samples were then thoroughly mixed with the solution. After completing this reaction, the absorbances were read at 450 nm. A standard curve was always generated with each assay run. Intra-assay coefficients of variation for all the samples ranged from 0.24% to 8.92%. The minimum detectable concentration of MDA-protein adducts in plasma was set to be 6 pmol/mL (Cell Biolabs, Inc.).

Reduced glutathione (GSH). GSH was measured in the blood using the Human reduced glutathione (GSH) ELISA Kit (MyBioSource, San Diego, CA, USA) following the specific manufacturer's instructions and previously described methodologies [17,18]. Fifty microL samples were added to every sample well, and incubated with horseradish peroxidase (HRP)-conjugate reagent at 37°C for 60 minutes. The plate was covered with a closure plate membrane during the experiment. After four washes, 50 microL chromogen solution A and 50 microL chromogen solution B were added to each well and samples were then incubated at 37ºC in the dark for 15 minutes. Finally, 50 microL of the stop solution were poured into each well. The absorbance in each sample was read at 450 nm. Intra-assay coefficients for all

the samples ranged from 0.07% to 8.69%. The minimum detectable concentration of GSH in plasma was set to be 1.56 μmol/L (MyBioSource).

Plasma levels of Trolox Equivalent Antioxidant Capacity (TEAC). TEAC levels were determined using the OxiSelect™ Trolox Equivalent Antioxidant Capacity (TEAC Assay Kit (ABTS, Cell Biolabs, Inc., San Diego, CA, USA) following the manufacturer's instructions. Twenty-five microL samples were added to the microplate well, and upon addition of 150 microL of the diluted 2,2′-azino-bis (3-ethylbenzothiazoline-6-sulfonic acid) reagent, samples were mixed thoroughly. Samples were then incubated on an orbital shaker for five minutes. Finally, the absorbance was read at 405 nm in all the sample wells. Antioxidant activity was determined by comparison with the Trolox standards. Intra-assay coefficients of variation for all the samples ranged from 0.39% to 9.96%. (Cell Biolabs, Inc.). The minimum detectable concentration of TEAC in plasma was set to be 250.29 g/mol (Cell Biolabs, Inc).

Quantification of myeloperoxidase in plasma. Plasma levels of myeloperoxidase were measured using the Human Myeloperoxidase ELISA Kit (MyBioSource, San Diego, CA, USA) following the specific manufacturer's instructions and previously described methodologies [17,18]. First, one hundred microL-samples were added into each sample well. Samples were then covered with aluminium foil and were incubated at room temperature with gentle shaking for 90 minutes. After three washes, samples were incubated with one hundred microL Biotin-Labeled Detection Antibody Working Solution at 37°C for 60 minutes. After three more washes, one hundred microL Streptavidin-HRP Working solution were added to each well and incubated at 37°C for 45 minutes. After five additional washes, one hundred microL of TMB Substrate Solution were added and incubated at 37°C

in the dark for 30 minutes. Finally, one hundred microL Stop Solution were added into each well. The absorbance in each sample was read at 450 nm. Intra-assay coefficients for all the samples ranged from 0.10% to 6.88%. The minimum detectable concentration of myeloperoxidase in plasma was set to be 312 pg/mL (MyBioSource).

Quantification of cyclooxygenase in plasma. The Human Cyclooxygenase 2 ELISA Kit (MyBioSource, San Diego, CA, USA) was used following the specific manufacturer's instructions and previously described methodologies [17,18]. Briefly, 50 microL sample was added to each well. One hundred microL HRP-Conjugate Reagent was added to the samples that were covered with a Closure Plate Membrane, and were subsequently incubated at 37°C for 60 minutes. After four washes, 50 microL Chromogen Solution A and 50 microL Chromogen Solution B were added. Subsequently afterwards, samples were mixed gently to be incubated in the dark at 37°C for 15 minutes. Finally, 50 microL Stop solution was added to each well. The absorbance of each sample was read at 450 nm. Intra-assay coefficients for all the samples ranged from 1% to 9%. The minimum detectable concentration of cyclooxygenase 2 in plasma was set to be 0.1 ng/mL (MyBioSource).

REFERENCES

1. Martínez-García, M.Á.; Máiz, L.; Olveira, C.; Girón, R.M.; de la Rosa, D.; Blanco, M.; Cantón, R.; Vendrell, M.; Polverino, E.; de Gracia, J.; et al. Spanish Guidelines on the Evaluation and Diagnosis of Bronchiectasis in Adults. *Arch. Bronconeumol.* **2018**, *54*, 79–87, doi:10.1016/j.arbres.2017.07.015.
2. Chalmers, J.D.; Chang, A.B.; Chotirmall, S.H.; Dhar, R.; McShane, P.J. Bronchiectasis. *Nat. Rev. Dis. Prim.* **2018**, *4*, 45, doi:10.1038/s41572-018-0042-3.
3. Martínez-García, M.A.; De Gracia, J.; Relat, M.V.; Girón, R.M.; Carro, L.M.; De La Rosa Carrillo, D.; Olveira, C.; Martinez-Garcia, M.A.; De Gracia, J.; Vendrell Relat, M.; et al. Multidimensional approach to non-cystic fibrosis bronchiectasis: The FACED score. *Eur. Respir. J.* **2014**, *43*, 1357–1367, doi:10.1183/09031936.00026313.
4. Martinez-Garcia, M.A.; Athanazio, R.A.; Girón, R.; Máiz-Carro, L.; de la Rosa, D.; Olveira, C.; de Gracia, J.; Vendrell, M.; Prados-Sánchez, C.; Gramblicka, G.; et al. Predicting high risk of exacerbations in bronchiectasis: the E-FACED score. *Int. J. Chron. Obstruct. Pulmon. Dis.* **2017**, *12*, 275–284, doi:10.2147/COPD.S121943.
5. Chalmers, J.D.; Goeminne, P.; Aliberti, S.; McDonnell, M.J.; Lonni, S.; Davidson, J.; Poppelwell, L.; Salih, W.; Pesci, A.; Dupont, L.J.; et al. The Bronchiectasis Severity Index. An International Derivation and Validation Study. *Am. J. Respir. Crit. Care Med.* **2014**, *189*, 576–585, doi:10.1164/rccm.201309-1575OC.
6. Aliboni, L.; Pennati, F.; Gelmini, A.; Colombo, A.; Ciuni, A.; Milanese, G.; Sverzellati, N.; Magnani, S.; Vespro, V.; Blasi, F.; et al. Detection and Classification of Bronchiectasis through Convolutional Neural Networks. *J. Thorac. Imaging* **2021**,

Publish Ah, doi:10.1097/RTI.0000000000000588.

7. Sancho-Muñoz, A.; Guitart, M.; Rodríguez, D.A.; Gea, J.; Martínez-Llorens, J.; Barreiro, E. Deficient muscle regeneration potential in sarcopenic COPD patients: Role of satellite cells. *J. Cell. Physiol.* **2021**, *236*, 3083–3098, doi:10.1002/jcp.30073.
8. Puig-Vilanova, E.; Martínez-Llorens, J.; Ausin, P.; Roca, J.; Gea, J.; Barreiro, E. Quadriceps muscle weakness and atrophy are associated with a differential epigenetic profile in advanced COPD. *Clin. Sci.* **2015**, *128*, 905–921, doi:10.1042/CS20140428.
9. Puig-Vilanova, E.; Ausin, P.; Martinez-Llorens, J.; Gea, J.; Barreiro, E. Do Epigenetic Events Take Place in the Vastus Lateralis of Patients with Mild Chronic Obstructive Pulmonary Disease? *PLoS One* **2014**, *9*, e102296, doi:10.1371/journal.pone.0102296.
10. Puig-Vilanova, E.; Rodriguez, D.A.; Lloreta, J.; Ausin, P.; Pascual-Guardia, S.; Broquetas, J.; Roca, J.; Gea, J.; Barreiro, E. Oxidative stress, redox signaling pathways, and autophagy in cachectic muscles of male patients with advanced COPD and lung cancer. *Free Radic. Biol. Med.* **2015**, *79*, 91–108, doi:10.1016/j.freeradbiomed.2014.11.006.
11. Barreiro, E.; Salazar-Degracia, A.; Sancho-Muñoz, A.; Gea, J.; Salazar-Degracia, A.; Sancho-Muñoz, A.; Gea, J.; Salazar-Degracia, A.; Sancho-Muñoz, A.; Gea, J.; et al. Endoplasmic reticulum stress and unfolded protein response profile in quadriceps of sarcopenic patients with respiratory diseases. *J. Cell. Physiol.* **2019**, *234*, 11315–11329, doi:10.1002/jcp.27789.
12. Rodriguez, D.A.; Kalko, S.; Puig-Vilanova, E.; Perez-Olabarría, M.; Falciani, F.; Gea,

J.; Cascante, M.; Barreiro, E.; Roca, J. Muscle and blood redox status after exercise training in severe COPD patients. *Free Radic. Biol. Med.* **2012**, *52*, 88–94, doi:10.1016/j.freeradbiomed.2011.09.022.

13. Bhalla, M.; Turcios, N.; Aponte, V.; Jenkins, M.; Leitman, B.S.; McCauley, D.I.; Naidich, D.P. Cystic fibrosis: scoring system with thin-section CT. *Radiology* **1991**, *179*, 783–788, doi:10.1148/radiology.179.3.2027992.
14. Roberts, H.R. Airflow obstruction in bronchiectasis: correlation between computed tomography features and pulmonary function tests. *Thorax* **2000**, *55*, 198–204, doi:10.1136/thorax.55.3.198.
15. Murray, P.R.; Washington, J.A. Microscopic and baceriologic analysis of expectorated sputum. *Mayo Clin. Proc.* **1975**, *50*, 339–44.
16. EUCAST: AST of bacteria Available online: https://www.eucast.org/ast_of_bacteria/ (accessed on May 31, 2021).
17. Mateu-Jiménez, M.; Sánchez-Font, A.; Rodríguez-Fuster, A.; Aguiló, R.; Pijuan, L.; Fermoselle, C.; Gea, J.; Curull, V.; Barreiro, E. Redox Imbalance in Lung Cancer of Patients with Underlying Chronic Respiratory Conditions. *Mol. Med.* **2016**, *22*, 85–98, doi:10.2119/molmed.2015.00199.
18. Barreiro, E.; Fermoselle, C.; Mateu-Jimenez, M.; Sánchez-Font, A.; Pijuan, L.; Gea, J.; Curull, V. Oxidative stress and inflammation in the normal airways and blood of patients with lung cancer and COPD. *Free Radic. Biol. Med.* **2013**, *65*, 859–871, doi:10.1016/j.freeradbiomed.2013.08.006.

Table S2. Systemic inflammatory and oxidative stress parameters in bronchiectasis patients according to smoking history

	Healthy controls	Bronchiectasis patients	
		Non-smokers	Ex-smokers
	N=26	N=21	N=9
**Systemic inflammatory parameters,** $\bar{x}$ (SD)			
Total leukocytes, x 10^3/µL	6.3 (1.6)	6.4 (1.6)	6.3 (1.5)
Total neutrophils, x 10^3/µL	3.9 (1.2)	4.1 (1.5)	4.1 (1.0)
Neutrophils, %	57.6 (6.9)	62.4 (8.2)	65.1 (6.5)
Total lymphocytes, x 10^3/µL	2.1 (0.6)	1.5 (0.3)	1.4 (0.5)*
Lymphocytes, %	31.7 (6.3)	25.4 (6.4)***	22.4 (5.4)**
Total eosinophils, x 10^3/ µL	0.15 (0.12)	0.16 (0.09)**	0.16 (0.10)***
Eosinophils, %	2.3 (1.6)	2.6 (1.4)	2.4 (1.5)
Platelets, x 10^3/µL	246 (63)	264 (57)	243 (95)
CRP, mg/dL	0.23 (0.41)	0.62 (0.72)	0.92 (1.3)*
ESR, mm/h	8 (7)	16 (14)*	14 (11)
Fibrinogen, mg/dL	305 (69)	383 (72)**	339 (106)
Alpha-1 antitrypsin, mg/dL	117.9 (18.3)	127.7 (22.0)	143.7 (30.3)*
Ceruloplasmin, mg/dL	22.7 (5.1)	26.4 (4.1)	28.6 (7.8)*
IgE, IU/mL	44 (42)	67 (76)	64 (97)
IgG aspergillus, mg/L	26 (23)	35 (38)	41 (30)
IgM, mg/dL	96 (44)	90 (59)	163 (113)*,$^{\#}$
IgA, mg/dL	249 (131)	313 (123)	369 (159)
IgG, mg/dL	1089 (199)	1257 (292)	1313 (564)
Myeloperoxidase, pg/mL	1.11 10^5 (0.32 10^5)	1.34 10^5 (0.37 10^5)	1.28 10^5 (0.41 10^5)
Cyclooxygenase 2, ng/mL	0.43 (0.23)	0.38 (0.14)	0.57 (0.15)$^{\#}$
**Systemic oxidative stress markers,** $\bar{x}$ (SD)			
Oxidized DNA, ng/mL	16.7 (2.3)	17.4 (4.1)	16.8 (2.6)
MDA-protein adducts, pmol/mL	72.3 (28.9)	103.6 (31.5)*	95.6 (27.6)
GSH, µmol/L	8.2 (2.4)	8.7 (3.7)	15.3 (8.3)*,$^{\#}$
TEAC, µM	5.19 10^3 (0.11 10^3)	5.21 10^3 (0.07 10^3)	5.15 10^3 (0.14 10^3)

Values are presented as mean (standard deviation). Abbreviations: N, number; CRP, C-reactive protein; ESR, erythrocyte sedimentation rate; IgE, immunoglobulin E; IgG aspergillus, immunoglobulin G aspergillus; IgM, immunoglobulin M; IgA, immunoglobulin A; IgG, immunoglobulin G; MDA-protein adducts, malondialdehyde-protein adducts; GSH, reduced glutathione; TEAC, trolox equivalent antioxidant capacity; µL, microliter; mg, milligrams; mm, millimeters; h, hour; dL, deciliter; IU, international unit; L, liter; pg, picograms; mL, milliliter; ng, nanograms; pmol, picomole; µmol, micromole; µM, micromole. Statistical analyses and significance: *, $p \leq 0.05$; **, $p \leq 0.01$; ***, $p \leq 0.001$ between any study group of patients and the healthy controls; $^{\#}$, $p \leq 0.05$ between ex-smoker and non-smoker patients.

Table S3. Systemic inflammatory and oxidative stress parameters in bronchiectasis patients according to pseudomona aeruginosa colonization

	Healthy controls	Bronchiectasis patients	
		Non-pseudomona aeruginosa	Pseudomona aeruginosa
	N=26	N=21	N=9
**Systemic inflammatory parameters,** $\bar{x}$ (SD)			
Total leukocytes, x 10^3/µL	6.3 (1.6)	6.4 (1.5)	6.3 (1.9)
Total neutrophils, x 10^3/µL	3.9 (1.2)	4.1 (1.3)	4.01 (1.5)
Neutrophils, %	57.6 (6.9)	63.7 (7.7)*	62.0 (8.1)
Total lymphocytes, x 10^3/µL	2.1 (0.6)	1.52 (0.39)***	1.48 (0.41)**
Lymphocytes, %	31.7 (6.3)	24.6 (7.0)***	24.2 (3.8)**
Total eosinophils, x 10^3/ µL	0.15 (0.12)	0.15 (0.07)	0.20 (0.14)
Eosinophils, %	2.3 (1.6)	2.4 (1.1)	2.9 (2.0)
Platelets, x 10^3/µL	246 (63)	254 (63)	265 (86)
CRP, mg/dL	0.23 (0.4)	0.76 (1.07)*	0.60 (0.37)
ESR, mm/h	8 (7)	16 (14)*	13 (10)
Fibrinogen, mg/dL	305 (69)	370 (89)*	369 (75)
Alpha-1 antitrypsin, mg/dL	117.9 (18.3)	132.8 (26.7)	131.7 (23.7)
Ceruloplasmin, mg/dL	22.7 (5.1)	27.2 (6.3)*	26.7 (2.9)
IgE, IU/mL	44 (42)	57 (75)	85 (95)
IgG aspergillus, mg/L	26 (23)	37 (37)	36 (30)
IgM, mg/dL	96 (44)	95 (76)	150 (94)
IgA, mg/dL	249 (131)	304 (121)	390 (152)**
IgG, mg/dL	1089 (199)	1161 (259)	1535 (506)***,$^{\#\#}$
Myeloperoxidase, pg/mL	1.11 10^5 (0.32 10^5)	1.34 10^5 (0.4 10^5)	1.25 10^5 (0.4 10^5)
Cyclooxygenase 2, ng/mL	0.43 (0.23)	0.41 (0.15)	0.49 (0.20)
**Systemic oxidative stress markers,** $\bar{x}$ (SD)			
Oxidized DNA, ng/mL	16.7 (2.3)	17.0 (4.1)	17.7 (2.7)
MDA-protein adducts, pmol/mL	72.3 (28.9)	98.2 (26.6)*	108.5 (37.7)*
GSH, µmol/L	8.2 (2.4)	9.5 (6.1)	13.7 (5.5)*,$^{\#}$
TEAC, µM	5.19 10^3 (0.11 10^3)	5.2 10^3 (0.1 10^3)	5.2 10^3 (0.09 10^3)

Values are presented as mean (standard deviation). Abbreviations: N, number; CRP, C-reactive protein; ESR, erythrocyte sedimentation rate; IgE, immunoglobulin E; IgG aspergillus, immunoglobulin G aspergillus; IgM, immunoglobulin M; IgA, immunoglobulin A; IgG, immunoglobulin G; MDA-protein adducts, malondialdehyde-protein adducts; GSH, reduced glutathione; TEAC, trolox equivalent antioxidant capacity; µL, microliter; mg, milligrams; mm, millimeters; h, hour; dL, deciliter; IU, international unit; L, liter; pg, picograms; mL, millilitre; ng, nanograms; pmol, picomole; µmol, micromole; µM, micromole. Statistical analyses and significance: *, $p \leq 0.05$; **, $p \leq 0.01$; ***, $p \leq 0.001$ between any study group of patients and the healthy controls; $^{\#}$, $p \leq 0.05$; $^{\#\#}$, $p \leq 0.01$ between pseudomona aeruginosa group and non-pseudomona aeruginosa group of patients.
